# Association between pregnancy exposure to air pollution and birth weight in selected areas of Norway

**DOI:** 10.1186/s13690-016-0138-8

**Published:** 2016-06-29

**Authors:** Sviatlana Panasevich, Siri Eldevik Håberg, Geir Aamodt, Stephanie J. London, Hein Stigum, Wenche Nystad, Per Nafstad

**Affiliations:** Domain for Mental and Physical Health, Norwegian Institute of Public Health, Oslo, Norway; Department of Landscape Architecture and Spatial Planning, Norwegian University of Life Sciences, Ås, Norway; National Institute of Environmental Health Sciences, National Institutes of Health, Department of Health and Human Services, Research Triangle Park, North Carolina, USA; Department of General Practice and Community Medicine, Medical Faculty, University of Oslo, Oslo, Norway

**Keywords:** Air pollution, Birth weight, MoBa, The Norwegian Mother and Child Cohort Study

## Abstract

**Background:**

Exposure to air pollution has adverse effects on cardiopulmonary health of adults. Exposure to air pollution in pregnancy may affect foetal development. However, the evidence of such effect remains inconsistent. We investigated the effects of exposure to air pollution during pregnancy on birth outcomes.

**Methods:**

This study, based within the Norwegian Mother and Child Cohort Study (MoBa), includes 17,533 participants living in the two largest cities in Norway: Oslo and Bergen, and their two surrounding counties: Akershus and Hordaland. Air pollution levels at residential addresses were estimated using land use regression models and back-extrapolated to the period of each pregnancy using continuous monitoring station data. Birth outcomes were birth weight, low birth weight, gestational age, and preterm delivery obtained from the Medical Birth Registry of Norway. Information on lifestyle factors was collected from MoBa questionnaires completed by mothers during pregnancy. Linear and logistic regression models were used to analyse the associations between pregnancy NO_2_ exposure and birth outcomes.

**Results:**

We found a statistically significant negative association between pregnancy exposure to NO_2_ and birth weight −43.6 (95%CI −55.8 to −31.5) g per 10 μg/m^3^ NO_2_. However, after adjusting for either area or the combination of parity and maternal weight, no substantive effects of air pollution exposure were evident.

**Conclusions:**

Exposure to air pollution during pregnancy was associated with decrease in birth weight, but area-related and lifestyle factors attenuated this association. We found no statistically significant associations of air pollution exposure with gestational age, low birth weight or preterm delivery.

**Electronic supplementary material:**

The online version of this article (doi:10.1186/s13690-016-0138-8) contains supplementary material, which is available to authorized users.

## Background

Air pollution has been shown to have systemic effects including inflammation and oxidative stress [1]. The associations between exposure to higher air pollution levels and respiratory and cardiovascular morbidity in adults have been shown in numerous studies [1, 2]. There is increasing interest in the possibility that exposure to air pollution during pregnancy leads to developmental effects in the foetus, such as lower birth weight or shorter gestational age. Results of these relatively recent studies are inconsistent, and there are relatively few data on putative causative biological pathways, critical time windows, and confounding by socioeconomic factors [2, 3]. Exposure to air pollution during pregnancy may lead to health impairment later in life, including reduced lung function, increased respiratory morbidity and altered immune system maturation [4]. Adverse birth outcomes may be a harbinger of air pollution effects on further developmental changes that can only be detected later. The majority of studies associate reduced birth weight and low birth weight with exposure to CO, NO_2_, and/or particulate matter (PM_10_ and PM_2.5_) [5]. Our study investigated NO_2_ as one of the major traffic-related pollutants and also as an indicator of ambient air pollution in Norway.

With the development of new methods, epidemiologic studies have been able to provide more individualized exposure assessment by using dispersion or land use regression (LUR) models for estimating air pollution exposure at residential addresses. In this paper we built LUR models for traffic-related air pollutant NO_2_ for the specific areas of Norway that we studied: two city areas (Oslo and Bergen) and their two surrounding counties (Akershus and Hordaland). The selected areas represent the most populated areas in Norway and also include the territories with highest levels of ambient air pollution. Each of these four areas has different land use practices, road network density, industrial activities and landscape specifics. To our knowledge, previously the associations of modelled air pollution exposure with pregnancy outcomes were studied in Norway only in Oslo [6, 7]. In the study of Oslo alone, no association between term birth weight and traffic air pollution was seen [6]. Oslo data were included in the consortium European Study of Cohorts for Air Pollution (ESCAPE) which reported an association of pregnancy exposure to PM_2.5_ with an increased risk of low birthweight at term but no significant decrease in birth weight [7].

We investigated the effects of exposure to traffic-related air pollution during pregnancy in selected urban and county areas of Norway on birth outcomes, including birth weight and the length of gestation.

## Methods

### Study population

The Norwegian Mother and Child Cohort Study (MoBa) is a prospective population-based pregnancy cohort study conducted by the Norwegian Institute of Public Health [8, 9]. Participants were recruited from all over Norway from 1999 to 2008. Among invited women, 40.6 % consented to participate. The whole cohort includes 114,500 children, 95200 mothers and 75200 fathers. Participants living in Oslo, Akershus, Bergen and Hordaland with information on home address at the time of delivery were included in our study (*N* = 22149). We excluded consequently participants with missing pregnancy NO_2_ exposure information (*N* = 3876), multiple births (*N* = 664) and non-live births (*N* = 76). Only data on singleton live births were used in the analysis. Total number of participants from the studied areas with non-missing air pollution exposure data was 17533 (born from 2001 to 2009), of which 4669 from Oslo, 7554 from Akershus, 3869 from Bergen and 1441 from Hordaland (Table 1). Birth records were obtained from the Medical Birth Registry of Norway (MBRN). Mothers participating in the MoBa study filled a number of questionnaires. We used data on lifestyle characteristics from questionnaire 1 filled at recruitment (approximately at week 17–18 of pregnancy) and questionnaire 3 filled at week 30 of pregnancy. MoBa has approvals from the regional Ethics committee and the Norwegian Data Inspectorate. The current study is based on version VI of the quality-assured data files released for research on the 15^th^ April 2011.

**Table 1:** Descriptive statistics for study participants from the MoBa cohort: N (%) or mean ± standard deviation

	Oslo (*N* = 4669)	Akershus (*N* = 7554)	Bergen (*N* = 3869)	Hordaland (*N* = 1441)	Total (*N* = 17533)
Birth weight^a^, g	3539 ± 545	3617 ± 558	3592 ± 550	3628 ± 553	3592 ± 554
Low birth weight	153 (3.3)	192 (2.5)	107 (2.8)	39 (2.7)	491 (2.8)
Missing		7 (0.1)	3 (0.1)		10 (0.1)
Gestational age, weeks	39.7 ± 1.9	39.8 ± 1.9	39.8 ± 1.9	39.7 ± 1.9	39.8 ± 1.9
Preterm delivery	241 (5.2)	340 (4.5)	170 (4.4)	68 (4.7)	819 (4.7)
Women who changed address during pregnancy	629 (13.5)	1023 (13.5)	513 (13.3)	171 (11.9)	2336 (13.3)
Parity					
0	2819 (60.4)	3032 (40.1)	1930 (49.9)	529 (36.7)	8310 (47.4)
1	1424 (30.5)	3052 (40.4)	1314 (34.0)	538 (37.3)	6328 (36.1)
≥ 2	426 (9.1)	1470 (19.5)	625 (16.2)	374 (26.0)	2895 (16.5)
Birth weight by parity, g					
0	3471 ± 544	3512 ± 547	3484 ± 545	3524 ± 547	3492 ± 546
1	3645 ± 522	3676 ± 542	3687 ± 502	3652 ± 554	3669 ± 531
≥ 2	3630 ± 562	3712 ± 579	3722 ± 596	3742 ± 533	3706 ± 575
Sex of child					
Boy	2413 (51.7)	3808 (50.4)	1950 (50.4)	754 (52.3)	8925 (50.9)
Girl	2256 (48.3)	3746 (49.6)	1919 (49.6)	687 (47.7)	8608 (49.1)
Maternal age, years	31.3 ± 4.1	31.4 ± 4.5	30.5 ± 4.6	29.5 ± 4.8	31.0 ± 4.5
Marital status					
Married/cohabit	4530 (97.0)	7335 (97.1)	3576 (92.4)	1339 (92.9)	16780 (95.7)
Other	139 (3.0)	219 (2.9)	293 (7.6)	102 (7.1)	753 (4.3)
Maternal education					
Less than high school	140 (3.0)	495 (6.6)	207 (5.4)	144 (10.0)	986 (5.6)
High school	804 (17.2)	2062 (27.3)	842 (21.8)	467 (32.4)	4175 (23.8)
Up to 4 years of college	1734 (37.1)	2768 (36.6)	1455 (37.6)	523 (36.3)	6480 (37.0)
More than 4 years of college (master or professional degree)	1762 (37.7)	1772 (23.5)	1115 (28.8)	218 (15.1)	4867 (27.8)
Missing	229 (4.9)	457 (6.1)	250 (6.5)	89 (6.2)	1025 (5.9)
Maternal smoking during pregnancy	171 (3.7)	555 (7.4)	225 (5.8)	134 (9.3)	1085 (6.2)
Missing	221 (4.7)	443 (5.9)	247 (6.4)	89 (6.2)	1000 (5.7)
Maternal weight^a^, kg	65.3 ± 10.4	67.3 ± 11.4	66.3 ± 11.0	68.4 ± 11.9	66.7 ± 11.2
Maternal height^a^, cm	168.4 ± 6.1	168.3 ± 5.9	168.0 ± 5.9	167.9 ± 6.1	168.2 ± 5.9
Season of birth					
Winter	1042 (22.3)	1650 (21.8)	1015 (26.2)	392 (27.2)	4099 (23.4)
Spring	1224 (26.2)	2052 (27.2)	1020 (26.4)	390 (27.1)	4686 (26.7)
Summer	1292 (27.7)	2102 (27.8)	900 (23.3)	336 (23.3)	4630 (26.4)
Autumn	1111 (23.8)	1750 (23.2)	934 (24.1)	323 (22.4)	4118 (23.5)
LUR modelled NO_2_ exposure during pregnancy, μg/m^3^	21.6 ± 4.4	10.3 ± 3.8	13.2 ± 6.2	6.3 ± 4.3	13.6 ± 6.9

^a^Missing data: 10 (0.1 %) birth weight, 1487 (8.5 %) maternal weight, 1102 (6.3 %) maternal height. European Union air quality standard for NO_2_: 1-year average 40 μg/m^3^, *LUR* land use regression

### Outcomes and covariates

For assessing birth outcomes we used continuous MBRN variables birth weight (grams) and gestational age (weeks). Air pollution can potentially affect the size of developing foetus. Therefore estimating gestational age was based on the last menstrual periods as estimates based on ultrasound assessments are dependent on early foetal growth [10]. If information on the last menstrual period was missing (*N* = 718, 9.5 %), we used data from ultrasound measurements to determine gestational age. We also studied binary outcomes: low birth weight (<2500 g) and preterm delivery (<37 completed weeks of gestation).

The following characteristics were also extracted from the MBRN: sex of the child (boy; girl), parity (0; 1; ≥2), mother’s age at birth (years), marital status (married/cohabit; other). Questionnaire information was used to determine: maternal education (less than high school; high school; up to 4 years of college; more than 4 years of college (master or professional degree)), maternal smoking during pregnancy (never; any smoking during pregnancy), maternal weight at the beginning of pregnancy (kg), maternal height (cm). Exposure to air pollution was estimated at the registered address at delivery. Women who did not change their address during pregnancy were used in a sensitivity analysis. Area variable was defined by the location of the address at delivery: Oslo, Akershus, Bergen, Hordaland.

### Air pollution exposure

Estimating air pollution exposure during pregnancy was based on the methodology developed for the ESCAPE project [7, 11]. LUR models for NO_2_ levels were built for the studied areas in order to account for regional specifics. In the models we used air pollution measurements conducted in 2010 for Oslo and Akershus, and in 2011 for Bergen and Hordaland. Measurement campaigns included three rounds of approximately two weeks duration with NO_2_ measurements (during winter, summer and an intermediate season) within a one year period. Measurement sites were selected to represent the range of residential exposure for each study area. For the analysis we included sites with no missing data, and no geocoding mismatches.

The mean exposures over the three measurement periods were averaged to obtain a yearly mean NO_2_ level that was used for LUR modelling. LUR models were built separately for Oslo and Akershus. One model was built for Bergen and Hordaland together due to a low number of valid measurement sites for Hordaland. Predictors for building the LUR model were obtained from a GIS analysis of the N50 and VBASE maps (obtained in February 2013) providing information on land use, residential density, types of landscape and road network information. Predictor variables are listed in Additional file 1: Table S1. We used altitude, distances to roads, port, airport, and to sea, estimates of land use types within the buffers of 100, 300, 500, 1000 and 5000 m. We included all categories of roads except walking and bicycling paths. Major roads included only roads of European, state and county levels. The summarized lengths of roads were estimated within the buffers of 25, 50, 100, 300, 500 and 1000 m. We built multiple linear regression models and performed diagnostic model tests according to the method described by Beelen and colleagues [11]. Final models were validated for: multicollinearity between included variables (Variance Inflation Factors), influential observations (Cook’s D), heteroscedasticity and spatial autocorrelation (Moran’s I) of residuals to assess the independence assumption. We used leave-one-out cross-validation (LOOCV) method to evaluate model performance.

Yearly means of air pollution levels at residential address at birth were estimated using the resulting LUR models. Variables in models were truncated in accordance to the range of corresponding variables used for LUR model building. Negative modelled values were replaced with 0.01. In order to account for temporal variability, we used the ratio method of back-extrapolation to the period of each pregnancy using continuous routine monitoring station data [7]. Daily NO_2_ measurements were obtained from the Norwegian Institute for Air Research database “Luftkvalitet.info” for the period 2000–2012 in Oslo (used for Oslo and Akershus), and for the period 2003–2012 in Bergen (used for Bergen and Hordaland). The daily estimates of exposure were calculated as the LUR-modelled yearly estimate multiplied by the ratio between daily NO_2_ routine monitoring station measurement and an annual average for the year when LUR measurement campaign took place. Daily NO_2_ exposure estimates were averaged separately for 1^st^, 2^nd^, and 3^rd^ trimester, and also over the whole pregnancy. Exposures by trimester and the whole pregnancy exposure were highly correlated and therefore we decided to use one exposure estimate: the average NO_2_ exposure during the whole pregnancy.

### Statistical analysis

We used linear regression models to analyse associations between pregnancy NO_2_ exposure and birth weight and gestational age. Logistic regression models were used to evaluate associations between pregnancy NO_2_ exposure and low birth weight and preterm delivery. The results are presented for both crude and adjusted models for a 10 μg/m^3^ increase in NO_2_. Adjustment variables were selected based on the literature analysis. Model 1 is adjusted for: maternal education, birth season, sex of child, maternal age, maternal marital status, maternal smoking during pregnancy, and maternal height. Model 2 contains Model 1 adjustment plus the area variable. Model 3 contains Model 1 adjustment plus parity and maternal weight. We performed stratified analysis by area (Oslo, Akershus, Bergen, Hordaland), smoking status of the mother during pregnancy, parity, sex of child, maternal education level, and season of birth in order to explore potential effect modifiers. Sensitivity analysis of the results was performed in a subgroup of women who did not change address during pregnancy and in a subgroup of women for whom gestational age was determined from the last menstrual period – as a preferable method for assessment of potential effects of air pollution on the size of the developing foetus [10]. Additional analysis was performed in a subset of participants with no missing data. We used ArcGIS10.1 software (Esri, CA, USA) for GIS analyses; statistical analyses were performed using STATA 13.0 (StataCorp, Texas, USA).

## Results

Overall birth weight was lower in cities (Oslo and Bergen) and higher in the two county areas (Akershus and Hordaland), however, these differences were less prominent when comparing birth weight within each parity strata (Table 1). Having had at least two children prior to the study pregnancy was more common in Hordaland (26 %) than in Oslo (9.1 %). Educational differences were also notable with the highest proportion of higher education in Oslo. Maternal smoking during pregnancy was uncommon (<10 %) throughout but highest in Hordaland.

The LUR models created for estimating NO_2_ yearly means are presented in Table 2. For Oslo, the important predictors were forested and semi-natural areas within the buffer of 1000 m and inverse squared distance to major road. The important predictors in the Akershus NO_2_ model were area of water in the buffer of 500 m, distance to major road, and agricultural areas within the buffer of 1000 m. The model for levels of NO_2_ in Bergen and Hordaland included the following: lengths of major roads within the buffers 50 m, 50 to 100 m, 100 to 1000 m, inverse squared distance to port, high and low density residential land within the buffer of 1000 m, altitude, agricultural area within the buffer of 1000 m, and distance to the sea. The adjusted R^2^ of the models ranged from 59 to 87 % (Table 2).

**Table 2:** Description of the developed land use regression models for NO_2_

Study area	LUR model^a^	R^2^ of model	Adjusted R^2^ of model	RMSE (cross-validation) (μg m^−3^)	Number of sites	Moran’s I (*p*-value)	Measured concentration (μg/m^3^)^b^
Oslo	24.59 – 8.40E-06*NATURAL1000 + 9684.22*DISTINVMAJOR2	67 %	61 %	5.3	14	−0.09 (0.45)	19.1 [2.8 – 30.0]
Akershus	15.79 + 0.000042*WATER500 - 0.01*DISTMAJOR - 3.79E-06*AGRO1000	59 %	55 %	4.5	36	0.01 (0.41)	13.7 [5.3 – 30.3]
Bergen and Hordaland	7.38 + 0.01*MAJORLENGTH50_100 + 859649.5*DISTINVPORT2 + 4.20E-06*HLDRES1000 + 0.05*MAJORLENGTH50 + 0.0003*MAJORLENGTH100_1000 - 0.05*ALT – 0.00003*AGRO1000 + 0.002*DISTSEA	87 %	85 %	4.3	46	−0.12 (0.11)	18.7 [6.8 – 51.0]

^a^See Additional file 1: Table S1 for definitions of the variable names. ^b^Mean [min - max]. *LUR* land use regression

The resulting estimates for pregnancy exposure to NO_2_ were created by applying LUR model to each residential address at birth and back-extrapolating in accordance with individual pregnancy timing. The mean pregnancy exposure to NO_2_ in the entire study population was 13.6 μg/m^3^, with regionally-specific highest estimate in Oslo (21.6 μg/m^3^), and lowest in Hordaland county (6.3 μg/m^3^) (Table 1). The mean exposures during pregnancy were below the European Union standard of average 1 year exposure of 40 μg/m^3^ NO_2_.

**Fig. 1:**
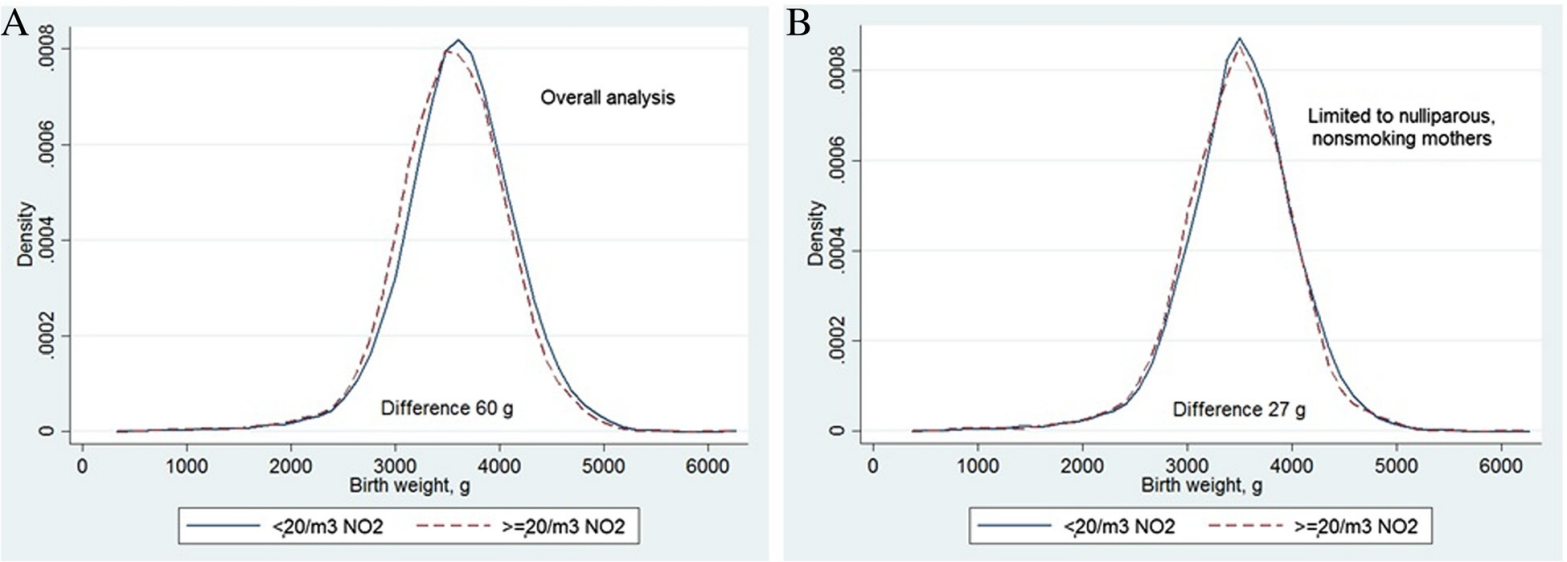
Distribution of birth weight according to pregnancy exposure to NO_2_. Comparing groups with mean pregnancy exposures to NO_2_ below 20 μg/m^3^ and above or equal 20 μg/m^3^. The differences were significant (t-test) in both groups: overall analysis (A) 60 g (*p* < 0.0001); and analysis limited to nulliparous, nonsmoking mothers (B) 27 g (*p* = 0.047)

Comparing the overall birth weight distributions, we found a shift towards lower birth weight in children with pregnancy exposure to NO_2_ above or equal to 20 μg/m^3^ (corresponding to the highest quartile of exposure in our study population) compared to those exposed to below 20 μg/m^3^ NO_2_, with estimated 60 g difference in means (*p*< 0.0001) between these groups (Fig. 1a). The same comparison of birth weight distributions among first-borns in non-smoking mothers gave us a smaller difference of 27 g (*p* = 0.047) (Fig. 1b). In the adjusted regression Model 1 we found a statistically significant negative association between pregnancy exposure to NO_2_ and birth weight −43.6 (95%CI −55.8 to −31.5) g per 10 μg/m^3^ NO_2_ (Table 3). The estimate changed very little in sensitivity analysis restricted to women who did not change their address during pregnancy, and in an analysis restricted to women for whom gestational age was estimated with last menstrual period data. However, the effect estimate was strongly attenuated and no longer statistically significant in further adjustment (Models 2 and 3). Among the model covariates, those with the strongest effect in attenuating the air pollution effect estimates were either study area (Model 2) or a combination of parity and maternal weight (Model 3) (Table 3). Results of the analyses repeated in a subset of participants with no missing data (*N* = 15829) did not change substantially from the results in the main analyses.

**Table 3:** Main and stratified analysis of association between pregnancy exposure to NO_2_ and birth weight

	Crude			Model 1^a^			Model 2^b^			Model 3^c^		
	*N*	Beta 95 % CI	*p*-value	*N*	Beta 95 % CI	*p*-value	*N*	Beta 95 % CI	*p*-value	N	Beta 95 % CI	*p*-value
Main analysis												
Entire study population	17523	−37.9 (−49.7 to −26.0)	<0.001	16273	−43.6 (−55.8 to −31.5)	<0.001	16273	−5.6 (−23.6 to 12.4)	0.54	15829	−7.4 (−19.6 to 4.8)	0.24
Women who did not change address	15191	−37.4 (−50.2 to −24.7)	<0.001	14196	−42.7 (−55.7 to −29.6)	<0.001	14196	−7.0 (−26.3 to 12.3)	0.48	13818	−4.7 (−17.8 to 8.4)	0.48
LMP-based GA only	16805	−35.4 (−47.5 to −23.2)	<0.001	15618	−40.8 (−53.3 to −28.4)	<0.001	15618	−3.2 (−21.6 to 15.1)	0.73	15195	−5.8 (−18.3 to 6.7)	0.36
Stratified analysis												
Oslo	4669	7.5 (−27.7 to 42.7)	0.68	4380	−5.9 (−42.8 to 31.0)	0.75				4285	12.5 (−24.3 to 49.3)	0.51
Akershus	7547	10.5 (−22.8 to 43.9)	0.54	6982	8.9 (−25.4 to 43.1)	0.61				6759	29.2 (−4.8 to 63.1)	0.09
Bergen	3866	−15.6 (−43.7 to 12.4)	0.28	3577	−4.8 (−33.0 to 23.4)	0.74				3490	19.8 (−7.7 to 47.2)	0.16
Hordaland	1441	−37.6 (−104.6 to 29.4)	0.27	1334	−36.0 (−103.5 to 31.5)	0.30				1295	−26.7 (−92.7 to 39.2)	0.43
Not smoking	15440	−41.3 (−53.8 to −28.8)	<0.001	15229	−43.3 (−55.8 to −30.8)	<0.001	15229	−6.6 (−25.1 to 12.0)	0.49	14835	−5.6 (−18.2 to 6.9)	0.38
Smoking	1083	−28.3 (−80.0 to 23.3)	0.28	1044	−45.5 (−97.7 to 6.8)	0.09	1044	22.1 (−51.8 to 96.1)	0.56	994	−27.3 (−80.1 to 25.5)	0.31
Parity 0	8304	−16.8 (−33.3 to −0.4)	0.045	7803	−17.8 (−34.7 to −1.0)	0.04	7803	4.3 (−20.5 to 29.0)	0.74	7594	−8.3 (−25.2 to 8.5)	0.33
Parity 1	6326	−0.6 (−20.6 to 19.4)	0.95	5858	−6.9 (−27.4 to 13.5)	0.51	5858	21.8 (−8.2 to 51.8)	0.15	5695	2.0 (−18.3 to 22.4)	0.85
Parity ≥2	2893	−26.5 (−60.3 to 7.4)	0.13	2612	−31.0 (−66.4 to 4.4)	0.09	2612	17.8 (−31.7 to 67.4)	0.48	2540	−24.8 (−59.9 to 10.4)	0.17
Boys	8921	−30.7 (−47.5 to −13.8)	<0.001	8290	−39.6 (−57.0 to −22.2)	<0.001	8290	−7.5 (−33.0 to 18.1)	0.57	8040	−5.4 (−22.8 to 12.1)	0.55
Girls	8602	−45.5 (−62.0 to −29.1)	<0.001	7983	−47.8 (−64.8 to −30.8)	<0.001	7983	−3.6 (−28.9 to 21.8)	0.78	7789	−9.4 (−26.4 to 7.6)	0.28
Education less than high school	985	−35.4 (−95.3 to 24.5)	0.25	968	−24.5 (−83.4 to 34.5)	0.42	968	−18.4 (−96.7 to 60.0)	0.65	905	−27.8 (−87.2 to 31.5)	0.36
Education high school	4173	−31.9 (−58.5 to −5.3)	0.02	4098	−36.0 (−62.3 to −9.7)	0.007	4098	10.4 (−27.3 to 48.1)	0.59	3948	4.8 (−21.7 to 31.3)	0.72
Education up to 4 years of college	6474	−42.4 (−61.5 to −23.3)	<0.001	6403	−44.0 (−62.8 to −25.3)	<0.001	6403	−1.5 (−30.2 to 27.1)	0.92	6262	−4.9 (−23.7 to 13.9)	0.61
Education more than 4 years of college (master or professional degree)	4866	−48.2 (−69.6 to −26.9)	<0.001	4804	−50.2 (−71.4 to −29.0)	<0.001	4804	−17.8 (−49.4 to 13.8)	0.27	4714	−13.3 (−34.5 to 8.0)	0.22
Born in winter	4097	−20.2 (−46.6 to 6.2)	0.13	3797	−35.3 (−62.5 to −8.2)	0.01	3797	7.8 (−31.1 to 46.7)	0.69	3677	4.9 (−22.4 to 32.1)	0.73
Born in spring	4684	−60.6 (−82.2 to −39.0)	<0.001	4355	−60.2 (−82.2 to −38.3)	<0.001	4355	−46.7 (−79.5 to −13.8)	0.005	4226	−28.5 (−50.6 to −6.4)	0.01
Born in summer	4626	−35.1 (−57.4 to −12.8)	0.002	4272	−40.5 (−63.3 to −17.6)	0.001	4272	14.2 (−20.7 to 49.1)	0.43	4167	−2.7 (−25.7 to 20.3)	0.82
Born in autumn	4116	−28.8 (−54.9 to −2.7)	0.03	3849	−31.9 (−58.6 to −5.3)	0.02	3849	16.1 (−23.0 to 55.1)	0.42	3759	5.1 (−21.4 to 31.7)	0.70

Effect estimate in grams per 10 μg/m^3^ NO_2_

*GA* gestational age, *LMP* last menstrual period

^a^Model 1 adjusted for: maternal education, birth season, sex of child, maternal age, maternal marital status, maternal smoking during pregnancy, maternal height

^b^Model 2 adjusted for: maternal education, birth season, sex of child, maternal age, maternal marital status, maternal smoking during pregnancy, maternal height, area

^c^Model 3 adjusted for: maternal education, birth season, sex of child, maternal age, maternal marital status, maternal smoking during pregnancy, maternal height, parity, maternal weight; in stratified analysis the corresponding stratification variable is not included in the adjustment

Upon stratification of the crude and the adjusted Model 1 by area, none of the associations of pregnancy air pollution exposure and birth weight were statistically significant (Table 3). The association of air pollution with birth weight was statistically significant in the much larger group of non-smokers but not the small group of smokers although associations for both groups were in the same direction. Inverse associations were seen at all levels of maternal education but were strongest in the groups with higher education. In the adjusted Models 2 and 3, where the overall findings were attenuated towards null, significant inverse associations between NO_2_ and birth weight were seen only in children born in spring (Table 3).

In our analysis we found no significant association of NO_2_ exposure during pregnancy and either gestational age, low birth weight or preterm delivery (Additional file 1: Table S2, Table S3 and Table S4).

## Discussion

We found a significant association of exposure to NO_2_ during pregnancy with lower birth weight. However, this association was strongly attenuated and no longer statistically significant when adjusting for study area. Adjusting for parity and maternal weight also substantially attenuated the association. No statistically significant associations were found for gestational age, low birth weight and preterm delivery.

Birth weight is associated with a variety of different factors: genetic and ethnic background, parity, maternal weight, socio-economic factors, medical conditions before or during pregnancy, and maternal lifestyle variables, most notably smoking during pregnancy [12]. The role of traffic-related exposure to NO_2_ during pregnancy have been investigated in a number of studies with inconsistent results. An association of NO_2_ exposure with low birth weight was found in the studies of Bell et al. 2007 (exposure during 1^st^ trimester), Lee et al. 2003 (exposure between months 3 and 5) [13, 14]. However, studies by Madsen et al. 2010, Liu et al. 2003, Aguilera et al. 2009, and Pedersen et al. 2013 did not find such association, [6, 7, 15, 16] even though the mean levels of exposure were comparable in these studies.

**Fig. 2:**
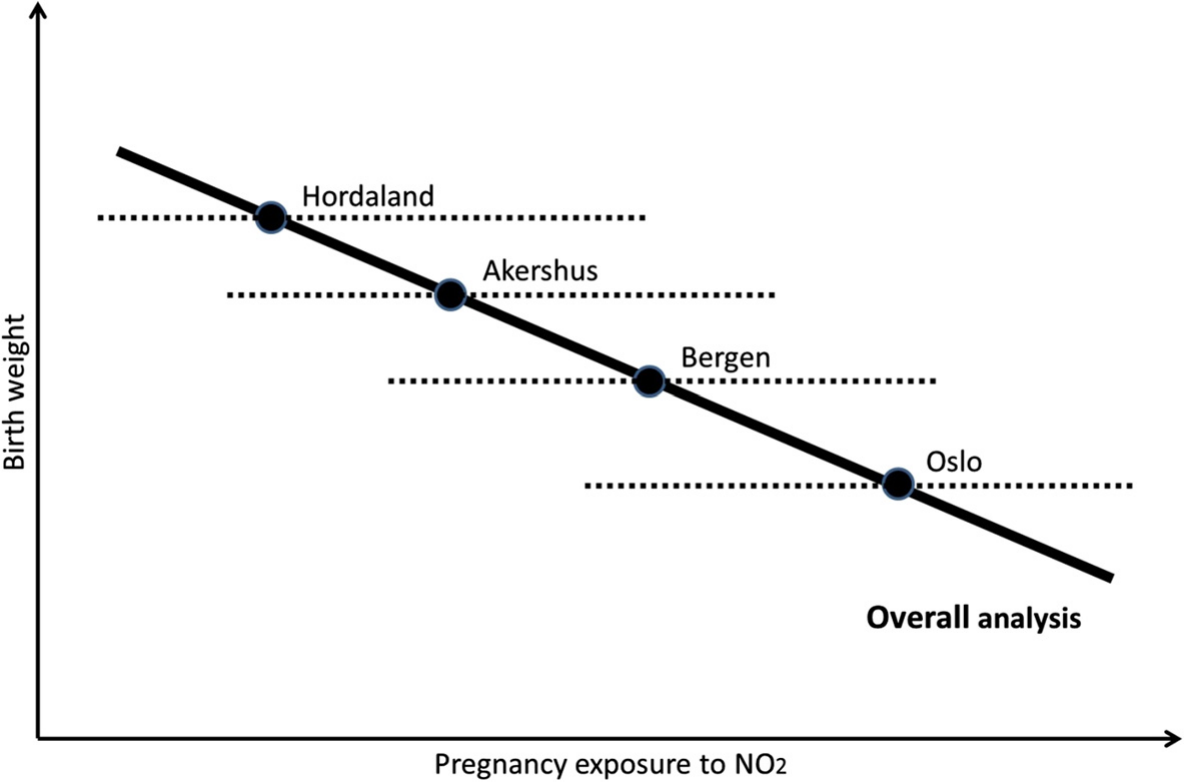
Simplified schematic representation of the association between air pollution and birth weight in the overall analysis (solid line) and individual analyses by area (dash lines). The lines are fitted lines of association, and the circles represent centroids for mean birth weight and mean pregnancy air pollution exposure by area

In our study, the association between pregnancy exposure to NO_2_ and birthweight was attenuated and no longer statistically significant when adjusting for study area. Our area variable may reflect the spatial distribution of air pollution, representing by itself the proxy of exposure, or it may represent unmeasured factors in each study area which also influence birth weight and pregnancy length. The area means for modelled NO_2_ range from highest in Oslo to lowest in Hordaland (Table 1), thus adjusting for area may remove some of the effect of spatially distributed air pollution [17]. Also, we found no association of pregnancy exposure to air pollution with decrease in birth weight in a stratified analysis by study area. The phenomenon where overall analysis indicates a strong reverse association between air pollution and birth weight and no such effect in the stratified analysis by area is theoretically presented as a scheme in Fig. 2. The observed effect might be due to minor methodological differences in initial measurement campaigns and their timing in different areas, differences in LUR models and in factors determining the NO_2_ levels on local scale. Areas may differ by mean levels of air pollution exposure, as well as by socioeconomic and lifestyle characteristics. For instance, social classes may be differently distributed within polluted areas depending on a region [4]. In the light of these area differences within one country with relatively homogenous population, more attention should be given to exploring socioeconomic and lifestyle differences in large multicenter studies and consortia [18].

Among the potential area-specific factors that attenuate the association of birth weight with air pollution exposure, our study singled out the combination of parity and maternal weight. These factors may be related to lifestyle, socioeconomic situation, and health state of mothers, and can vary by study area. Specifically in Norway, families with more than one child tend to move out from large cities, which may explain the difference in distribution of parity by study area. Stronger associations of air pollution exposure with adverse birth outcomes have been reported in less economically advantaged classes [19, 20]. In our data we observed a more pronounced decrease in birth weight in cities compared to counties and in mothers with higher levels of education compared to mothers with lower levels of education (Table 3). More travel to and from work and to other activities, visiting central more polluted parts of the city by higher educated women and city dwellers may result in a potential exposure misclassification [21].

Our study found no association of NO_2_ exposure during pregnancy with gestational age and preterm delivery. This is in line with other studies also reporting no association of NO_2_ exposure with preterm delivery [16, 22].

The important strengths of our study include standardized exposure assessment, detailed information on potential confounders, and a large study population. We highlighted the importance of area-specific factors that may confound the association between air pollution exposure and pregnancy outcomes. Our results suggest that these factors should be given a proper consideration in large multicenter studies. Further research is needed for identifying potential critical windows of exposure. In our data, exposures by trimester were highly correlated with whole pregnancy exposure. We assessed exposure at residence, but for more detailed individual exposure it is advisable to consider estimating exposures during travel and frequency of visits to city center. Most of our study participants did not move during pregnancy, but we did note a difference in distribution of multiparous women in our study areas, with higher proportion of them living in counties, and not in the cities. Besides the identification of the important confounders, such as parity and maternal weight, we might still have the residual confounding of unmeasured lifestyle factors in our data.

## Conclusions

In this large pregnancy cohort with detailed information on lifestyle and pregnancy factors, land use regression modelled exposure to NO_2_ during pregnancy was associated with a decrease in birth weight in some models. However, when adjusting for area-related and lifestyle factors this association was attenuated. We found no statistically significant associations of air pollution exposure with gestational age, low birth weight and preterm delivery.

## Additional file

Additional file 1:
**Table S1.** Predictor variables used in land use regression with variable names, units, buffer sizes, transformations and predefined direction of effect. **Table S2.** Main and stratified analysis of association between pregnancy exposure to NO_2_ and gestational age. **Table S3.** Main and stratified analysis of association between pregnancy exposure to NO_2_ and low birth weight. **Table S4.** Main and stratified analysis of association between pregnancy exposure to NO_2_ and preterm delivery. (DOCX 43 kb)

## Abbreviations

GA: gestational age
LMP: last menstrual period
LUR: land use regression
MBRN: Medical Birth Registry of Norway
MoBa: Norwegian Mother and Child Cohort Study
