# Supplementary material for: Association between pregnancy exposure to air pollution and birth weight in selected areas of Norway
Source: Arch Public Health. 2016 Jun 29;74:26. doi: 10.1186/s13690-016-0138-8 (PMC4926306; doi:10.1186/s13690-016-0138-8)
Supplement: Additional file 1: — Table S1. Predictor variables used in land use regression with variable names, units, buffer sizes, transformations and predefined direction of effect. Table S2. Main and stratified analysis of association between pregnancy exposure to NO2 and gestational age. Table S3. Main and stratified analysis of association between pregnancy exposure to NO2 and low birth weight. Table S4. Main and stratified analysis of association between pregnancy exposure to NO2 and preterm delivery. (DOCX 43 kb) [file 13690_2016_138_MOESM1_ESM.docx]

**SUPPLEMENT**

Supplement Table S1. Predictor variables used in land use regression with variable names, units, buffer sizes, transformations and predefined direction of effect.

| **Predictor variable** | **Name** | **Unit** | **Buffer** | **Transformation** | **Direction of effect** |
| --- | --- | --- | --- | --- | --- |
| High density residential land | HDRES | m^2^ | 100, 300, 500, 1000, 5000 |  | + |
| Low density residential land | LDRES | m^2^ | 100, 300, 500, 1000, 5000 |  | + |
| Sum high density and low density residential land | HLDRES | m^2^ | 100, 300, 500, 1000, 5000 |  | + |
| Industry | INDUSTRY | m^2^ | 100, 300, 500, 1000, 5000 |  | + |
| Distance to port | DISTPORT  DISTINVPORT  DISTINVPORT2 | m  m^-1^  m^-2^ |  | Inverse distance  Inverse distance squared | -  +  + |
| Urban green | URBGREEN | m^2^ | 100, 300, 500, 1000, 5000 |  | - |
| Forested and semi-natural areas | NATURAL | m^2^ | 100, 300, 500, 1000, 5000 |  | - |
| Agricultural areas | AGRO | m^2^ | 100, 300, 500, 1000, 5000 |  | - |
| Sum of urban green and forested and semi-natural and agricultural areas | GREEN | m^2^ | 100, 300, 500, 1000, 5000 |  | - |
| Area of water | WATER | m^2^ | 100, 300, 500, 1000, 5000 |  | - |
| Altitude | ALT  SQRALT | m  m |  | Square root | -  - |
| Coordinate variables | XCOORD  YCOORD | m  m |  |  |  |
| Area indicator | KOMM |  |  |  |  |
| Distance to sea | DISTSEA  DISTINVSEA  DISTINVSEA2 | m  m^-1^  m^-2^ |  |  | +  -  - |
| Distance to the nearest road | DISTROAD  DISTINVROAD  DISTINVROAD2 | m  m^-1^  m^-2^ |  | Inverse distance  Inverse distance squared | -  +  + |
| Distance to the nearest major road | DISTMAJOR  DISTINVMAJOR  DISTINVMAJOR | m  m^-1^  m^-2^ |  | Inverse distance  Inverse distance squared | -  +  + |
| Road length of all roads in a buffer | ROADLENGTH | m | 25, 50, 100, 300, 500, 1000 |  | + |
| Road length of major roads in a buffer | MAJORLENGTH | m | 25, 50, 100, 300, 500, 1000 |  | + |
| Distance to airport | DISTAIR  DISTINVAIR  DISTINVAIR2 | m  m^-1^  m^-2^ |  | Inverse distance  Inverse distance squared | -  +  + |

Supplement Table S2. Main and stratified analysis of association between pregnancy exposure to NO_2_ and gestational age

|  | **Crude** | | |  | **Model 1*** | | |  | **Model 2**† | | |  | **Model 3**‡ | | |
| --- | --- | --- | --- | --- | --- | --- | --- | --- | --- | --- | --- | --- | --- | --- | --- |
|  | N | Beta 95%CI | p-value |  | N | Beta 95%CI | p-value |  | N | Beta 95%CI | p-value |  | N | Beta 95%CI | p-value |
| Entire study population | 17533 | -0.02 (-0.06 to 0.02) | 0.37 |  | 16283 | -0.03 (-0.07 to 0.02) | 0.24 |  | 16283 | 0.03 (-0.03 to 0.10) | 0.34 |  | 15839 | -0.02 (-0.07 to 0.02) | 0.35 |
| Women who did not change address | 15197 | -0.03 (-0.07 to 0.01) | 0.18 |  | 14202 | -0.04 (-0.09 to 0.01) | 0.10 |  | 14202 | 0.02 (-0.05 to 0.09) | 0.52 |  | 13824 | -0.04 (-0.08 to 0.01) | 0.14 |
| LMP-based GA only | 16815 | -0.01 (-0.06 to 0.03) | 0.54 |  | 15628 | -0.02 (-0.07 to 0.02) | 0.35 |  | 15628 | 0.04 (-0.03 to 0.10) | 0.28 |  | 15205 | -0.02 (-0.07 to 0.03) | 0.39 |
| Oslo | 4669 | -0.03 (-0.15 to 0.10) | 0.65 |  | 4380 | -0.09 (-0.22 to 0.05) | 0.21 |  |  |  |  |  | 4285 | -0.10 (-0.24 to 0.04) | 0.16 |
| Akershus | 7554 | 0.09 (-0.03 to 0.20) | 0.15 |  | 6989 | 0.11 (-0.01 to 0.23) | 0.08 |  |  |  |  |  | 6766 | 0.10 (-0.02 to 0.23) | 0.10 |
| Bergen | 3869 | 0.03 (-0.06 to 0.13) | 0.50 |  | 3580 | 0.03 (-0.07 to 0.13) | 0.60 |  |  |  |  |  | 3493 | 0.04 (-0.06 to 0.14) | 0.42 |
| Hordaland | 1441 | 0.06 (-0.17 to 0.30) | 0.59 |  | 1334 | 0.10 (-0.13 to 0.34) | 0.39 |  |  |  |  |  | 1295 | 0.10 (-0.14 to 0.34) | 0.41 |

Effect estimate in grams per 10µg/m^3^ NO_2_

GA – gestational age; LMP – last menstrual period

*Model 1 adjusted for: maternal education, birth season, sex of child, maternal age, maternal marital status, maternal smoking during pregnancy, maternal height

†Model 2 adjusted for: maternal education, birth season, sex of child, maternal age, maternal marital status, maternal smoking during pregnancy, maternal height, area

‡Model 3 adjusted for: maternal education, birth season, sex of child, maternal age, maternal marital status, maternal smoking during pregnancy, maternal height, parity, maternal weight

Supplement Table S3. Main and stratified analysis of association between pregnancy exposure to NO_2_ and low birth weight

|  | **Crude** | | | |  | **Model 1*** | | | |  | **Model 2**† | | | |  | **Model 3**‡ | | | |
| --- | --- | --- | --- | --- | --- | --- | --- | --- | --- | --- | --- | --- | --- | --- | --- | --- | --- | --- | --- |
|  | N total | LBW cases | OR 95%CI | p-value |  | N total | LBW cases | OR 95%CI | p-value |  | N total | LBW cases | OR 95%CI | p-value |  | N total | LBW cases | OR 95%CI | p-value |
| Entire study population | 17523 | 491 | 1.01 (0.89 to 1.15) | 0.86 |  | 16273 | 457 | 1.04 (0.90 to 1.19) | 0.60 |  | 16273 | 457 | 0.80 (0.65 to 0.98) | 0.03 |  | 15829 | 435 | 0.95 (0.82 to 1.10) | 0.51 |
| Women who did not change address | 15191 | 416 | 0.96 (0.84 to 1.11) | 0.61 |  | 14196 | 391 | 0.98 (0.84 to 1.13) | 0.76 |  | 14196 | 391 | 0.78 (0.62 to 0.97) | 0.03 |  | 13818 | 374 | 0.90 (0.77 to 1.05) | 0.19 |
| LMP-based GA only | 16805 | 463 | 1.00 (0.88 to 1.15) | 0.95 |  | 15618 | 431 | 1.03 (0.89 to 1.19) | 0.67 |  | 15618 | 431 | 0.78 (0.63 to 0.96) | 0.02 |  | 15195 | 412 | 0.96 (0.83 to 1.11) | 0.60 |
| Oslo | 4669 | 153 | 0.77 (0.54 to 1.08) | 0.13 |  | 4380 | 142 | 0.86 (0.59 to 1.25) | 0.42 |  |  |  |  |  |  | 4285 | 137 | 0.80 (0.54 to 1.18) | 0.27 |
| Akershus | 7547 | 192 | 0.67 (0.45 to 0.98) | 0.04 |  | 6982 | 180 | 0.65 (0.43 to 0.98) | 0.04 |  |  |  |  |  |  | 6759 | 172 | 0.64 (0.42 to 0.98) | 0.04 |
| Bergen | 3866 | 107 | 0.93 (0.67 to 1.28) | 0.64 |  | 3577 | 100 | 0.89 (0.63 to 1.25) | 0.50 |  |  |  |  |  |  | 3490 | 95 | 0.80 (0.56 to 1.14) | 0.22 |
| Hordaland | 1441 | 39 | 0.88 (0.41 to 1.87) | 0.74 |  | 1334 | 35 | 0.79 (0.35 to 1.79) | 0.58 |  |  |  |  |  |  | 1295 | 31 | 0.65 (0.27 to 1.57) | 0.33 |

Effect estimate in grams per 10µg/m^3^ NO_2_

GA – gestational age; LMP – last menstrual period

*Model 1 adjusted for: maternal education, birth season, sex of child, maternal age, maternal marital status, maternal smoking during pregnancy, maternal height

†Model 2 adjusted for: maternal education, birth season, sex of child, maternal age, maternal marital status, maternal smoking during pregnancy, maternal height, area

‡Model 3 adjusted for: maternal education, birth season, sex of child, maternal age, maternal marital status, maternal smoking during pregnancy, maternal height, parity, maternal weight

Supplement Table S4. Main and stratified analysis of association between pregnancy exposure to NO_2_ and preterm delivery

|  | **Crude** | | | |  | **Model 1*** | | | |  | **Model 2**† | | | |  | **Model 3**‡ | | | |
| --- | --- | --- | --- | --- | --- | --- | --- | --- | --- | --- | --- | --- | --- | --- | --- | --- | --- | --- | --- |
|  | N total | PD cases | OR 95%CI | p-value |  | N total | PD cases | OR 95%CI | p-value |  | N total | PD cases | OR 95%CI | p-value |  | N total | PD cases | OR 95%CI | p-value |
| Entire study population | 17533 | 819 | 1.05 (0.95 to 1.16) | 0.32 |  | 16283 | 763 | 1.07 (0.97 to 1.20) | 0.19 |  | 16283 | 763 | 0.96 (0.82 to 1.13) | 0.63 |  | 15839 | 733 | 1.03 (0.92 to 1.15) | 0.66 |
| Women who did not change address | 15197 | 704 | 1.05 (0.94 to 1.17) | 0.40 |  | 14202 | 661 | 1.08 (0.96 to 1.21) | 0.18 |  | 14202 | 661 | 0.96 (0.81 to 1.15) | 0.68 |  | 13824 | 635 | 1.03 (0.91 to 1.16) | 0.62 |
| LMP-based GA only | 16815 | 782 | 1.05 (0.95 to 1.17) | 0.32 |  | 15628 | 729 | 1.08 (0.97 to 1.21) | 0.16 |  | 15628 | 729 | 0.96 (0.81 to 1.13) | 0.60 |  | 15205 | 701 | 1.04 (0.93 to 1.16) | 0.53 |
| Oslo | 4669 | 241 | 0.89 (0.67 to 1.19) | 0.44 |  | 4380 | 228 | 1.00 (0.74 to 1.36) | 0.98 |  |  |  |  |  |  | 4285 | 222 | 0.96 (0.70 to 1.31) | 0.78 |
| Akershus | 7554 | 340 | 0.87 (0.65 to 1.17) | 0.36 |  | 6989 | 316 | 0.84 (0.62 to 1.15) | 0.28 |  |  |  |  |  |  | 6766 | 305 | 0.83 (0.61 to 1.14) | 0.26 |
| Bergen | 3869 | 170 | 1.06 (0.83 to 1.35) | 0.65 |  | 3580 | 158 | 1.00 (0.77 to 1.30) | 0.99 |  |  |  |  |  |  | 3493 | 150 | 0.93 (0.71 to 1.22) | 0.61 |
| Hordaland | 1441 | 68 | 1.43 (0.82 to 2.50) | 0.21 |  | 1334 | 61 | 1.16 (0.63 to 2.12) | 0.64 |  |  |  |  |  |  | 1295 | 56 | 1.11 (0.59 to 2.10) | 0.75 |

Effect estimate in grams per 10µg/m^3^ NO_2_

GA – gestational age; LMP – last menstrual period

*Model 1 adjusted for: maternal education, birth season, sex of child, maternal age, maternal marital status, maternal smoking during pregnancy, maternal height

†Model 2 adjusted for: maternal education, birth season, sex of child, maternal age, maternal marital status, maternal smoking during pregnancy, maternal height, area

‡Model 3 adjusted for: maternal education, birth season, sex of child, maternal age, maternal marital status, maternal smoking during pregnancy, maternal height, parity, maternal weight
